# CuO Nanoparticles Reduce Toxicity and Enhance Bioaccumulation of Cadmium and Lead in the Cells of the Microalgae *Desmodesmus communis*

**DOI:** 10.3390/ijms25179167

**Published:** 2024-08-23

**Authors:** Svetlana P. Chebotaryova, Peter A. Baranchikov, Olga V. Zakharova, Tatiana A. Kozlova, Yevhen I. Maltsev, Maxim S. Kulikovskiy, Gregory V. Grigoriev, Alexander A. Gusev

**Affiliations:** 1 Scientific and Educational Center for Environmental Science and Biotechnology, Derzhavin Tambov State University, 392020 Tambov, Russia; sweta-chebotarjova@yandex.ru (S.P.C.); petrovi4-98@yandex.ru (P.A.B.); olgazakharova1@mail.ru (O.V.Z.); k_tatiana15@yahoo.com (T.A.K.); bboykick@outlook.com (G.V.G.); 2 Department of Functional Nanosystems and High-Temperature Materials, National University of Science and Technology “MISIS”, 119991 Moscow, Russia; 3 Laboratory of Ecology, Institute of Natural and Technical Systems RAS, 354024 Sochi, Russia; 4 K.A. Timiryazev Institute of Plant Physiology RAS, IPP RAS, 127276 Moscow, Russia; ye.maltsev@gmail.com (Y.I.M.); max-kulikovsky@yandex.ru (M.S.K.)

**Keywords:** bioaccumulation of heavy metals, cadmium, CuO nanoparticles, lead, microalgae, nanobioremediation, oxidative stress, toxicity

## Abstract

The removal of pollutants, including heavy metals, from the aquatic environment is an urgent problem worldwide. Actively developing nanotechnology areas is becoming increasingly important for solving problems in the field of the remediation of aquatic ecosystems. In particular, methods for removing pollutants using nanoparticles (NPs) are proposed, which raises the question of the effect of a combination of NPs and heavy metals on living organisms. In this work, we investigated the role of CuO-NPs in changing the toxicity of Cd and Pb salts, as well as the bioaccumulation of these elements in a culture of the microalga *Desmodesmus communis*. It was found that CuO-NPs at concentrations of 10, 100, and 1000 µg L^−1^ had no effect on the viability of microalgae cells. On the 14th day of the experiment, Cd at a concentration of 1 mg L^−1^ reduced the viability index by 30% and, when combined with CuO-NPs, by 25%, i.e., CuO-NPs slightly reduced the toxic effect of Cd. At the same time, in this experiment, when CuO-NPs and Cd were used together, the level of oxidative stress increased, including on the first day in mixtures with 1 mg L^−1^ Cd. Under the influence of Pb, the cell viability index decreased by 70% by the end of the experiment, regardless of the metal concentration. The presence of CuO-NPs slightly reduced the toxicity of Pb in terms of viability and reactive oxygen species (ROS). At the same time, unlike Cd, Pb without NPs caused ROS production on the first day, whereas the addition of CuO-NPs completely detoxified Pb at the beginning and had a dose-dependent effect on mixtures at the end of the experiment. Also, the introduction of CuO-NPs slightly reduced the negative effect of Pb on pigment synthesis. As a molecular mechanism of the observed effects, we prioritized the provocation of oxidative stress by nanoparticles and related gene expression and biochemical reactions of algae cells. Analysis of the effect of CuO-NPs on the Cd and Pb content in microalgae cells showed increased accumulation of heavy metals. Thus, when algae were cultured in an environment with Cd and CuO-NPs, the Cd content per cell increased 4.2 times compared to the variant where cells were cultured only with Cd. In the case of Pb, the increase in its content per one cell increased 6.2 times when microalgae were cultured in an environment containing CuO-NPs. Thus, we found that CuO-NPs reduce the toxic effects of Cd and Pb, as well as significantly enhance the bioaccumulation of these toxic elements in the cells of *D. communis* microalgae. The results obtained can form the basis of technology for the nanobioremediation of aquatic ecosystems from heavy metals using microalgae.

## 1. Introduction

Due to their unique physico-chemical properties, engineering nanoparticles (NPs) have high potential for both industrial and domestic use [1,2,3]. According to manufacturers’ reports, more than 1300 commercial consumer products, such as cosmetics, paints, food containers, sports equipment, biosensors, and catalysts, contain NPs [4].

One of the most widespread applications of NPs is their use as sorbents for cleaning polluted environments, including the removal of heavy metals [5,6,7]. Zero-valent nanosized iron is successfully used for soil and groundwater recultivation [8,9]. The effectiveness of Fe_3_O_4_ NPs for the adsorption of lead ions from water [10], ZnO NPs for the adsorption of copper ions [11], and TiO_2_, MgO, and Al_2_O_3_ NPs for the removal of cadmium, copper, nickel, and lead ions from aqueous solutions [12] has been demonstrated. At the same time, due to their sorption properties, NPs can play the role of carriers facilitating the entry of pollutants into living organisms (the “Trojan horse effect”), which can lead to an increase in the toxicity of pollutants [13]. Similar effects have been demonstrated for TiO_2_ NPs on the accumulation of toxic metals in fish [14,15] and plants [16]. However, in contrast, other studies demonstrated that NPs, such as TiO_2_ and SiO_2_, reduce metal toxicity in algae [17], higher plants [18,19], and mollusks [20].

Such contradictory results may be due to differences in the concentration of metals and/or NPs in the physiology of the tested organisms, the physico-chemical environmental conditions, and other factors. This indicates the need for a systematic and in-depth study of the effect of NPs on the toxicity and bioaccumulation of heavy metals.

Nanoscale CuO particles, one of the most widely used NPs, are often offered as antibacterial agents as alternatives to antibiotics, nanocomposite coatings, catalysts, lubricants, and agricultural pesticides [21,22]. However, there are relatively few studies focusing on the effectiveness of copper oxide NPs as a modifier for the microalgal bioremoval of hazardous toxicants from the aquatic environment, as well as the possible effects of these NPs as a “Trojan horse” enhancing the penetration and toxicity of other toxins.

In aquatic ecosystems, microscopic algae, as primary producers, are an easy target for most pollutants. A number of papers have been published showing the effects of various types of NPs, including CuO, on aquatic biota, including microalgae [23,24,25]. In our previous work, it was shown that *Desmodesmus* sp. is relatively tolerant to the impact of CuO-NPs, while the ICP-MS method revealed increased bioaccumulation of copper by microalgae cells [26].

It should be taken into account that nanoparticles are present in the aquatic environment in the form of complex mixtures with other pollutants, such as antibiotics, pesticides, heavy metals, etc., which can lead to synergism, sensitization, additivity, or antagonism of their action. Among heavy metals, As, Hg, Cr, Pb, and Cd are of the greatest concern [27].

Despite the widespread and proven significant environmental impact of heavy metals and NPs, our understanding of the effects caused by the combination of heavy metals and NPs on biological systems is still insufficient. Also relevant is the problem of developing and improving methods for the bioremoval of various pollutants, including NPs and heavy metals, from the environment. Microalgae play a key role in the ecophysiology of aquatic ecosystems and can also serve as biosorbents for the retention of pollutants [28,29,30]. However, to date, insufficient research has been conducted on the effect of NPs, specifically CuO-NPs, on the physiology of microalgae, particularly in the presence of heavy metals. 

Thus, we hypothesize that copper oxide nanoparticles can promote the bioremoval of toxic metals from aquatic environments by microalgae cells. Therefore, the goal of this work was to determine the toxic effects and specificity of the bioaccumulation of Cd and Pb in the cells of the microalgae *D. communis* in the presence of CuO-NPs.

## 2. Results

### 2.1. Strain Description

*D. communis* MZ–Ch293 (Chlorophyceae)

MORPHOLOGICAL DESCRIPTION: Coenobia of (two), four, or eight linearly cells. Cells are 8.0–16.2 µm long and 3.3–5.9 µm wide and elongated into a cylindrical shape. Internal cells have rounded apices and teeth. Marginal cells have narrowed, retracted, and slightly curved apices and linearly symmetrical main spines. The outermost cell wall layer between cell poles is often visible.

HABITAT: The strain MZ–Ch293 was isolated from a planktonic community near the beach of the Kagalnik River (46°56′7.57″ N, 39°39′38.20″ E), Rostov region, Russia, 12 July 2021.

SEQUENCE DATA: GenBank accession PQ165045 for the 18S rRNA gene partial sequence.

MOLECULAR ANALYSIS: Phylogenetic analysis (ML and BI methods) shows that *D. communis* MZ–Ch293 with other *Desmodesmus* strains formed a unified *Desmodesmus* clade with maximum statistical support (likelihood bootstrap 100 and posterior probability 1.0) within Chlorophyceae. *D. communis* MZ–Ch293 was most similar to the *D. communis* CCAP 258/266 and *D. communis* CCAP 276-4B strains.

### 2.2. Nanoparticle Assessment

**Figure 1:**
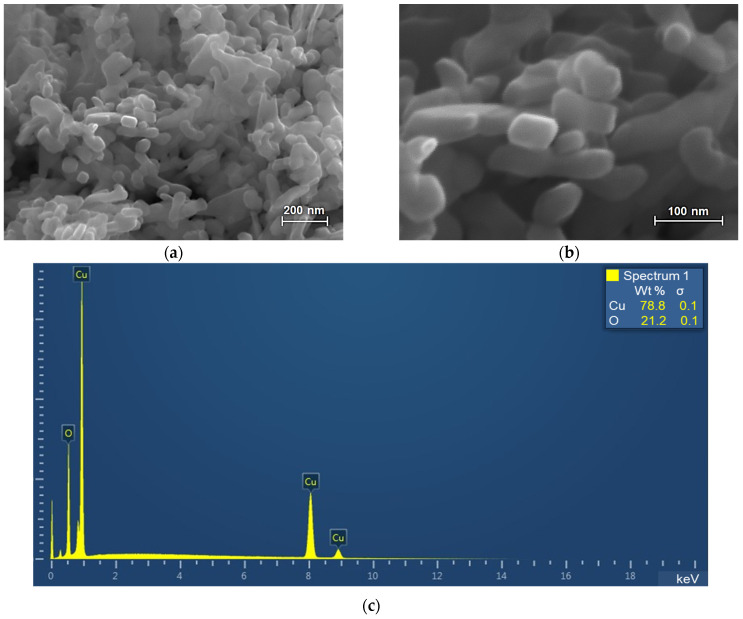
Scanning electron microscopy (SEM) analysis of the CuO nanoparticle (NP) sample: (**a**,**b**) micrographs; (**c**) energy dispersive X-ray spectroscopy.

During the CuO-NP study, it was found that the particles used in the work are rod-shaped structures with rounded ends assembled into aggregates (Figure 1a,b).

As can be seen from the presented microphotographs, the average diameter of individual particles is about 40–50 nm, and the length is 50–100 nm. Energy-dispersive X-ray spectroscopy (EDXA) shows that the analyzed powder is copper oxide without any impurities (Figure 1c). Thus, the electron microscopic examination shows the nanometer size of CuO particles (40–100 nm) and the absence of impurities in the sample.

**Figure 2:**
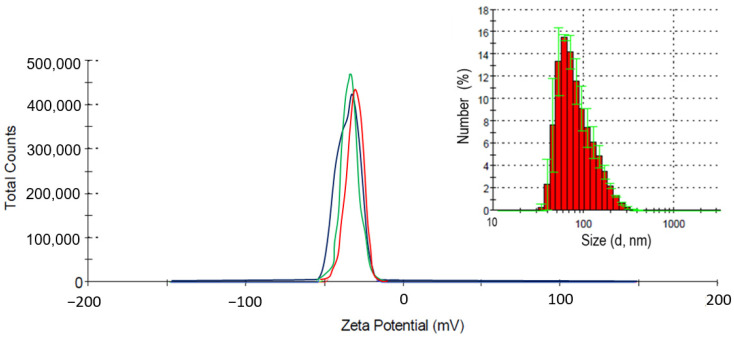
Zeta (ζ) potential and the dispersed composition of CuO in NP suspension.

The measurement of the ζ-potential of a suspension with a concentration of 500 µg L^−1^ nanoparticles shows a value of −34.4 mV (Figure 2), which indicates a high stability of the dispersed system.

The average particle size in the solution was 40–100 nm, which is consistent with the electron microscopy data. In addition, as can be seen from the presented dispersed composition, ultrasonic treatment effectively coped with large accumulations of particles; the maximum size of the aggregates, according to dynamic light scattering data, was no more than 200 nm (Figure 2 insert). Lines of different colors correspond to the results of three independent measurements.

### 2.3. The Effect of NPs and Heavy Metals on Microalgae

#### 2.3.1. Cell Viability

**Figure 3:**
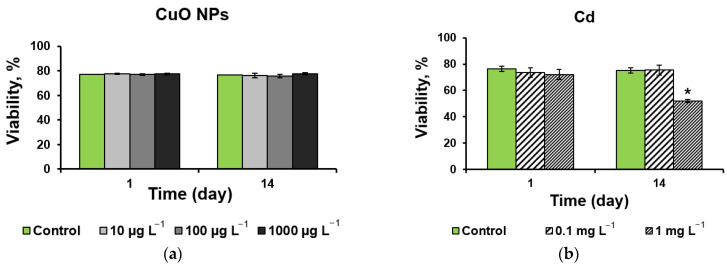

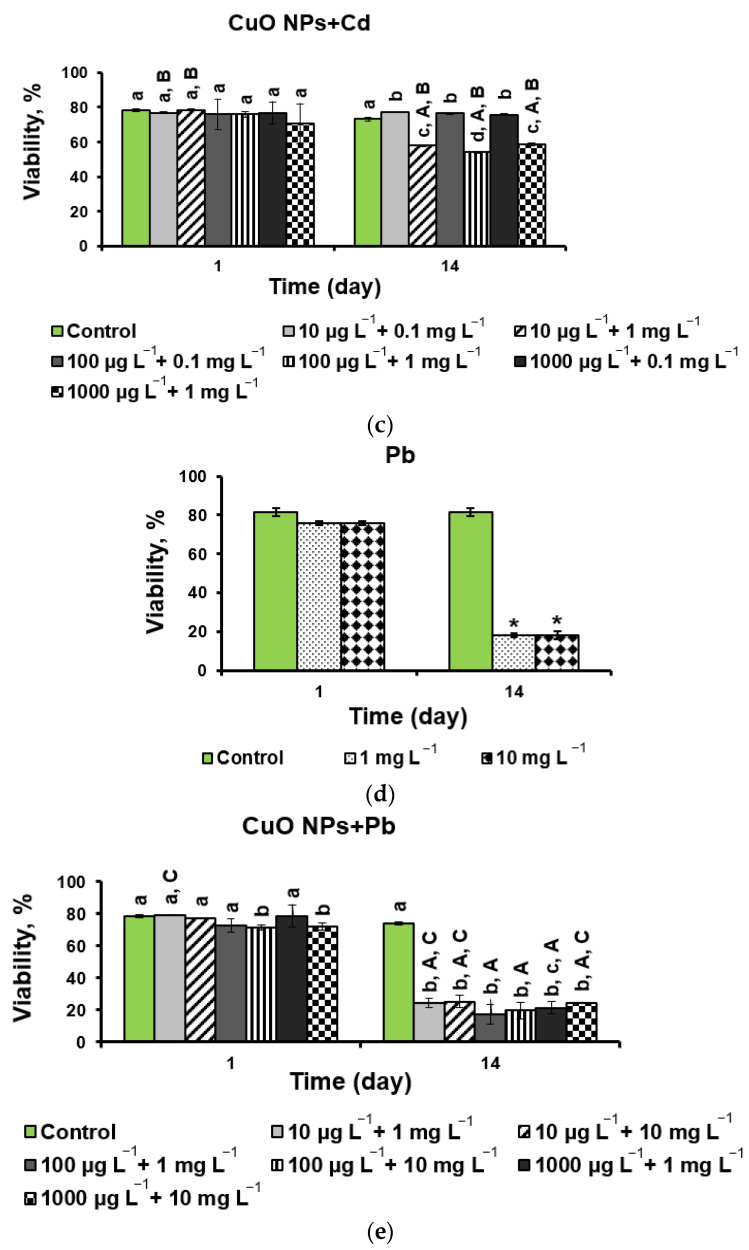
Viability of *D. communis* incubated with the addition of (**a**) CuO-NPs; (**b**) Cd;(**c**) CuO-NPs and Cd; (**d**) Pb; (**e**) CuO-NPs and Pb. The diagrams show the average values and standard deviations. * Significant differences with the control. Lowercase letters indicate statistically significant differences in the group (day 1 and day 14). Capital letters (A, B, C) demonstrate significant differences between two-component mixtures and single-component mixtures with corresponding concentrations of pollutants (CuO, Cd, and Pb, respectively) (*p* < 0.05).

The assessment of the effect of CuO-NPs on the viability of a *D. communis* culture shows the absence of a significant effect from the NPs in the selected concentrations (Figure 3a). At the same time, when microalgae with 1 mg L^−1^ Cd were exposed to the nutrient medium, a decrease of 30% was noted on the 14th day of the experiment (Figure 3b).

At the same concentration of Cd (1 mg L^−1^), by the end of the experiment (14 d), the viability index decreased by 25% with the combined use of CuO-NPs and Cd (Figure 3c). In mixtures of any concentration of NPs with 0.1 mg L^−1^ Cd, statistically significant stimulation of the viability of *D. communis* cells was noted compared with the control (Figure 3c).

As can be seen from Figure 3d, the introduction of Pb into the cultivation medium reduced the viability of *D. communis* by 75% by the end of the experiment regardless of the concentration of Pb. Similar data were obtained when microalgae were cultivated in a medium containing a mixture of CuO-NPs and Pb (Figure 3e). However, it should be noted that by the end of the experiment, three CuO-NPs + Pb mixtures (10 µg L^−1^ + 1 mg L^−1^, 10 µg L^−1^ + 10 mg L^−1^, and 1000 µg L^−1^ + 10 mg L^−1^) showed an increase in cell viability compared with the influence of Pb only (Figure 3d,e).

Almost all mixtures in this experiment reduced cell viability compared to the effect of NPs alone by the end of the experiment (Figure 3a,e).

#### 2.3.2. Oxidative Stress

**Figure 4:**
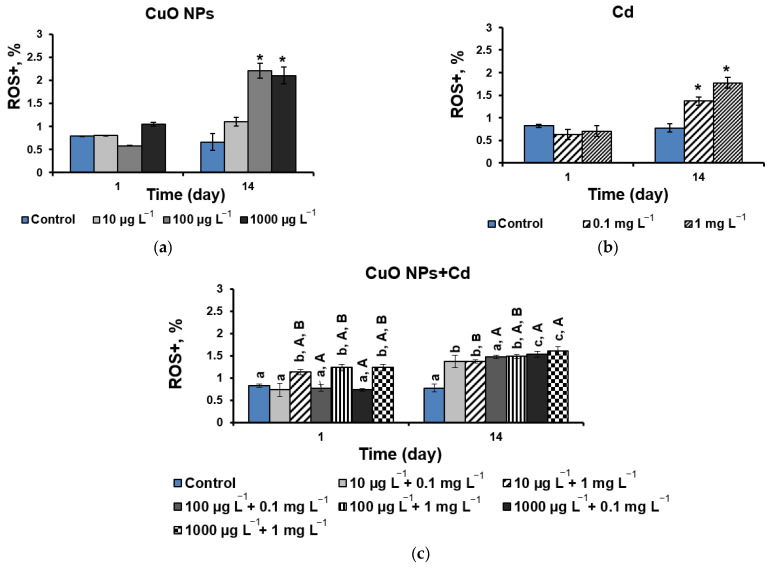

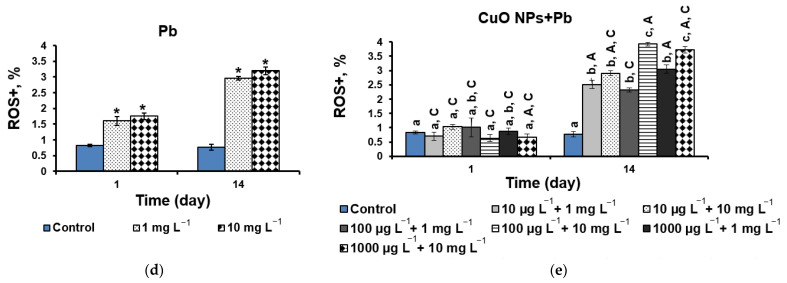
The level of oxidative stress of *D. communis* cells incubated with the addition of (**a**) CuO-NPs; (**b**) Cd; (**c**) CuO-NPs and Cd; (**d**) Pb; (**e**) CuO-NPs and Pb. * Significant differences with the control. Lowercase letters indicate statistically significant differences in the group (day 1 and day 14). Capital letters (A, B, C) demonstrate significant differences between two-component mixtures and single-component mixtures with corresponding concentrations of pollutants (CuO, Cd, and Pb, respectively) (*p* < 0.05). “ROS+” is the proportion of cells with oxidative stress.

The analysis of oxidative stress shows that, despite the absence of a significant effect of CuO-NPs on the viability of the *D. communis* culture, the cells were exposed to reactive oxygen species on the 14th day at 100 and 1000 µg L^−1^ NPs, with a 70% increase in reactive oxygen species (ROS) compared with the control (Figure 4a).

The addition of both tested Cd concentrations also activated ROS production by an average of 50% on the 14th day of the experiment while maintaining high cell viability at 0.1 mg L^−1^ Cd (Figure 3b and Figure 4b). When cells were exposed to mixtures of NPs and Cd, an increase in ROS production was noted on the first day for variants with a concentration of Cd 1 mg L^−1^ (Figure 4c) despite high cell viability for both components of the mixture (Figure 3a,b). Moreover, in these mixtures, there was a statistically significant increase in ROS production compared to the same concentrations of NPs without metal. However, by the end of the experiment (14 d), almost all mixtures (except 10 µg L^−1^ + 0.1 mg L^−1^ with no effect) showed a significant decrease in ROS formation compared with its formation in the presence of only NPs (Figure 4a,c). Compared with the individual effect of Cd, ROS production was suppressed in the mixtures (CuO-NPs + Cd: 10 µg L^−1^ + 1 mg L^−1^ and 100 µg L^−1^ + 1 mg L^−1^), whereas in other cases, no effect was observed.

When the effect of Pb on ROS production was investigated, a two-fold increase in the level of oxidative stress was observed on the first day of the experiment, and, after 14 days, the indicator increased by an average of four times (Figure 4d). On the first day of cell exposure in all NPs + Pb mixtures, ROS production significantly decreased compared to the effect of lead alone and remained unchanged compared to the effect of pure NPs. On day 14, the ROS values were more than three times higher than the control values for all treatment options (Figure 4e), and unlike mixtures with Cd, NPs + Pd mixtures showed a statistically significant increase in ROS formation compared to pure NPs (except for the CuO-NPs + Pb mixture at 100 µg L^−1^ + 1 mg L^−1^ without effect). The negative effect of Pb was significantly reduced or remained unchanged in most mixtures. An increase in ROS production was observed only in mixtures of 10 mg L^−1^ Pb with high concentrations of NPs (100 and 1000 µg L^−1^).

#### 2.3.3. Pigment Composition

**Figure 5:**
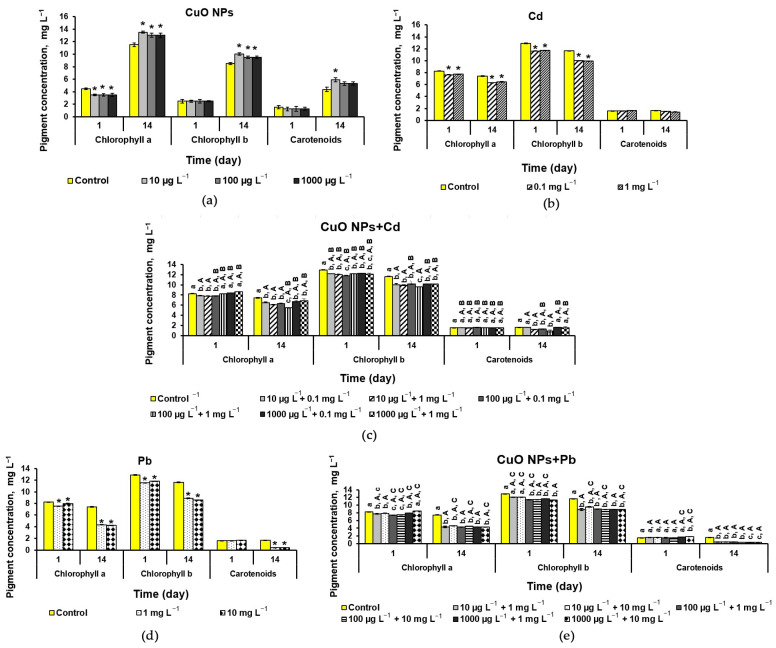
Pigment content in *D. communis* culture during cultivation with the addition of (**a**) CuO-NPs; (**b**) Cd; (**c**) CuO-NPs and Cd; (**d**) Pb; (**e**) CuO-NPs and Pb. * Significant differences with the control. Lowercase letters mean statistically significant differences in the group (day 1 and day 14). Capital letters (A, B, C) demonstrate significant differences between two-component mixtures and single-component mixtures with corresponding concentrations of pollutants (CuO, Cd, and Pb, respectively) (*p* < 0.05).

In the quantitative analysis of pigment composition in the case of microalgae cultivation with CuO-NPs, a statistically significant decrease in the content of chlorophyll-a (Chl-*a*) was noted on the first day of exposure, with a further increase in concentration by 12–15% by the end of the experiment when compared to the control. The content of chlorophyll-b (Chl-*b*) and carotenoids (CRs) also increased by 10–15% and 26%, respectively, by the end of the experiment. It is important to note that the greatest effect of the stimulation of all three pigments was observed at the lowest concentration of CuO-NPs at 10 µg L^−1^ (Figure 5a).

The addition of Cd inhibited the synthesis of chlorophylls after 5 h of exposure (Figure 5b). When NPs and Cd were added to the culture medium, the negative effect of heavy metal was significantly reduced; no inhibition of chlorophylls was observed on the first day of the experiment. However, the biosynthesis of CRs decreased (Figure 5c). Moreover, on the 14th day, when CuO was added at the maximum concentration, an increase in the content of Chl-*a* and Chl-*b* was also observed. The biosynthesis of carotenoids in mixtures depended on the concentration of Cd: a low Cd content (0.1 mg L^−1^) significantly reduced the toxicity of the metal, whereas a high one (1 mg L^−1^), in contrast, increased toxicity.

In the case of Pb, there was also a decrease in indicators, and for chlorophylls, this was observed on the first day, while the concentration of CRs did not change (Figure 5d). At the end of the experiment, the concentration of Chl-*a* decreased by 40% and Chl-*b* by 25%, regardless of the amount of Pb in the culture medium. The carotenoid content decreased by four times relative to the control. As can be seen from Figure 5d,e, the addition of CuO-NPs significantly reduced the negative effect of Pb on the first day of the experiment at a low concentration (1 mg L^−1^) and increased at a high concentration (10 mg L^−1^) for Chl-*a*, whereas the content of Chl-*b* increased in almost all mixtures, and CRs increased in mixtures with a maximum concentration of NPs. At the end of the experiment, the concentrations of Chl-*a* and Chl-*b* increased in mixtures with 10 mg L^−1^ Pb, while the CR content remained comparable to the action of a metal without NPs.

#### 2.3.4. Bioaccumulation Analysis

At the end of the experiment, the effect of CuO-NPs on the bioaccumulation of Cd and Pb in microalgae cells was analyzed. The results of the analysis of the accumulation of heavy metals are presented in Table 1.

Both mixtures of 1000 µg L^−1^ CuO-NPs with Cd and Pb stimulated the growth of the *D. communis* population. The addition of CuO-NPs to the culture medium increased the number of microalgae cells by an average of 1.6 times compared with the exposure of the culture with Cd and Pb alone at the end of the experiment.

The intracellular accumulation of metals increased in mixtures compared to exposure to metals alone, which is especially pronounced in the case of Pb. The presence of CuO-NPs contributed to a 1.5-fold increase in Cd content in crude biomass and a 1.05-fold increase in Cd content per cell by the end of the experiment compared with metal-only exposure.

In the case of Pb, a similar effect was observed. CuO-NPs led to a two-fold increase in Pb concentration in crude biomass and a 6.2-fold increase in heavy metal content per cell when microalgae were cultured in an environment with Pb and CuO-NPs.

## 3. Discussion

During the experiment, it was found that CuO-NPs in the studied concentrations did not affect the viability of microalgae cells despite the presence of oxidative stress for cells on day 14 at concentrations of 100 and 1000 µg L^−1^ (Figure 3a). Cadmium (Cd) at a concentration of 1 mg L^−1^ reduced the viability index by 30% (Figure 3b) and, when combined with nano-cupper, by 25% (Figure 3c), i.e., CuO-NPs slightly reduced the toxic effect of Cd. A decrease in Cd toxicity with the introduction of NPs may be associated with an increase in antioxidative activity and increased regulation of genes associated with ROS uptake [31]. At the same time, the mechanism of action may vary depending on the type of test object, Cd concentration, and type of nanomaterial used [32]. Stimulation of the viability of *D*. *communis* cells in mixtures with 0.1 mg L^−1^ Cd indicates a possible synergy between the components of a mixture with low Cd concentrations. However, further research is necessary to clarify this issue.

At the same time, in this experiment, when CuO-NPs and 1 mg L^−1^ Cd were used together, the level of oxidative stress increased on the first day, followed by a decrease in ROS production in all mixtures except for in the combination of minimum concentrations of NPs and a metal (Figure 4c). There is evidence from other authors of a synergistic increase in ROS content in CuO-NP and Cd mixtures [33]. However, another study showed that ROS formation was accompanied by disrupted chlorophyll synthesis and the inhibition of cell growth [34]. In this case, under the influence of CuO-NPs, the negative effect of Cd on pigment synthesis decreased. Probably, the lack of negative effects from ROS formation is associated with the activation of some protective mechanisms in microalgal cells, including the synthesis of the antioxidative enzymes superoxide dismutase, catalase, peroxidase, and glutathione reductase, as well as non-enzymatic antioxidative molecules, such as phytochelatins, pigments, polysaccharides, and polyphenols [35]. In addition, studies have shown that the basal content of ROS is necessary for living organisms [36]. They serve as a set of safe signaling molecules that regulate many different biological processes, such as an increase in the accumulation of lipids in microalgae [37].

When Pb was applied to the growth medium, the cell viability index decreased by 70% after two weeks of the experiment, regardless of both the metal concentration and the CuO-NP content (Figure 3d,e). At the same time and unlike Cd, Pb without NPs caused ROS production as early as the first day of exposure, while when CuO-NPs were added, the parameters were at the control level after 5 h of exposure, and even a decrease in ROS content was observed in the mixture with the highest tested concentrations of pollutants (Figure 4d,e). Despite a significant drop in viability and an increase in ROS production compared to the control, by the end of the experiment, these parameters were better or did not change compared to the effect of Pb alone in the mixtures. There is evidence in the literature that copper can stimulate the production of various antioxidative enzymes or low molecular weight antioxidants to control ROS levels in cells. It was shown that the combined action of Cu and Pb contributed to an increase in the activity of superoxide dismutase [34]. In general, it is reported that with the combined action of heavy metals and NPs, a decrease in oxidative stress in microalgae can be observed due to the antioxidant properties of Cu and changes in the chemical and structural characteristics of metals, as well as improvements in the metabolic processes in algal cells [38].

The addition of CuO-NPs had a different effect on the toxicity of the two tested metals regarding pigment synthesis (Figure 5). It is interesting to note that for both metals, the vast majority of mixtures with NPs increased the chlorophyll content on the first day of exposure, whereas CR decreased in the case of Cd and mostly did not change with Pb compared with exposure to metals without NPs. 

By the end of the experiment, the effect on mixtures varies significantly, with some differences between pigments and some between metals. It can be said that for Pb, there was a greater decrease in toxicity in mixtures in terms of chlorophyll content than for Cd. As for carotenoids, inhibition of synthesis was noted at high Cd concentrations, and a lower one stimulated CR synthesis in mixtures, whereas, for Pb, almost no mixtures affected CR production. The obtained results of the stimulation of pigment biosynthesis in mixtures may also be associated with an increase in antioxidative activity in cells under the action of Cu [34] or the formation of metal complexes less bioavailable for microalgae [39].

Many factors can influence the degree of inhibition of various physiological parameters of microalgae under the action of pollutants. These include the type and concentration of heavy metals or nanoparticles, exposure time, microalgal strain, and cultivation conditions [38]. The presented research confirms that the effect on mixtures of metals with NPs depends on the nature of the metal, the concentrations of the components of the mixture, and the tested physiological parameters of the algae. As a molecular mechanism of the observed effects, we prioritized the provocation of oxidative stress by nanoparticles and related gene expression and biochemical reactions of algae cells.

Analysis of the effect of CuO-NPs on Cd and Pb accumulation by microalgae cells (Table 1) shows increased concentrations of heavy metals in the presence of NPs. The data obtained indicate an increased bioaccumulation of heavy metals at the maximum concentration of CuO-NPs tested. In 2007, Limbach et al. [40] spoke for the first time about the “Trojan horse” effect, which implies facilitated penetration of toxic molecules adsorbed on NPs into cells. Studies show that NPs can cause changes in the membrane structure and increase its porosity, thereby facilitating the penetration of pollutants [41]. Contact with NPs induces the formation of new pores in the membrane through the mechanisms of lipid peroxidation, making it more permeable and less selective [42,43]. In a study by Qian et al. [34], it was found that Cu ions at a concentration of 0.5 µM increased the bioaccumulation of Cd ions; however, the opposite effect was observed at a concentration of Cu ions of 1.5 µM. A decrease in Cd accumulation by plants under the action of Cu was also recorded in another work [39]. In the presented work, the dependence of the increase or decrease in metal toxicity on the composition of concentrations in the Me + NPs mixture was noted when the toxicity of Pb (according to the formation of ROS and cell viability) weakened, increased, or remained statistically equal compared with the single action of the same metal concentrations. However, Cd in mixtures of Me + NPs behaved differently, demonstrating a direct dependence of the decrease in cell viability on the concentration of metal but not on the concentration of NPs.

Thus, we can conclude that CuO-NPs reduce the toxic effect of Cd (more pronounced) and Pb, as well as significantly enhance the bioaccumulation of these toxic elements in the cells of D. communis microalgae. The results obtained can form the basis of technology for the nanobioremediation of aquatic ecosystems from heavy metals using microalgae.

## 4. Materials and Methods

### 4.1. Test Object

#### 4.1.1. Cultivation

A culture of single-celled green algae *D. communis*, strain MZ–Ch293, was taken from the algae collection of the molecular systematics of aquatic plants of the Timiryazev Institute of Plant Physiology of the Russian Academy of Sciences.

*D. communis* was cultured on BG-11 medium [44,45] in glass conical flasks with a capacity of 250 mL, under constant illumination with LED lamps of white daylight (480 µE/(m^2^ s)) and maintaining a temperature of 25 ± 1 °C and pH of 7.1 ± 0.2.

#### 4.1.2. Molecular Analysis

Total genomic DNA was isolated using molecular biology grade Chelex 100 Chelating Resin (Bio-Rad Laboratories, Hercules, CA, USA), according to the manufacturer’s protocol 2.2. The amplification of the highly variable V4 region of the 18S rRNA gene was performed, as described by Zimmermann et al. [46], using the primers D512 for and D978rev. The amplification was performed using premade polymerase chain reaction (PCR) mastermixes (ScreenMix by Evrogen, Moscow, Russia) with the following program: 5 min of denaturation at 95 °C, followed by 35 cycles of denaturation at 94 °C (30 s), annealing at 52 °C (30 s), and elongation at 72 °C (50 s), with a final extension at 72 °C (8 min). The PCR products were quantified on a 1% agarose gel stained with SYBR^TM^ Safe (Life Technologies, Carlsbad, CA, USA) and purified using a mixture of FastAP, 10× FastAP Buffer, Exonuclease I (Thermo Fisher Scientific, Waltham, MA, USA), and water. The purified amplification products were sequenced using the PCR primers in a Genetic Analyzer 3500 instrument (Applied Biosystems, Arlington, VI, USA).

**Figure 6:**
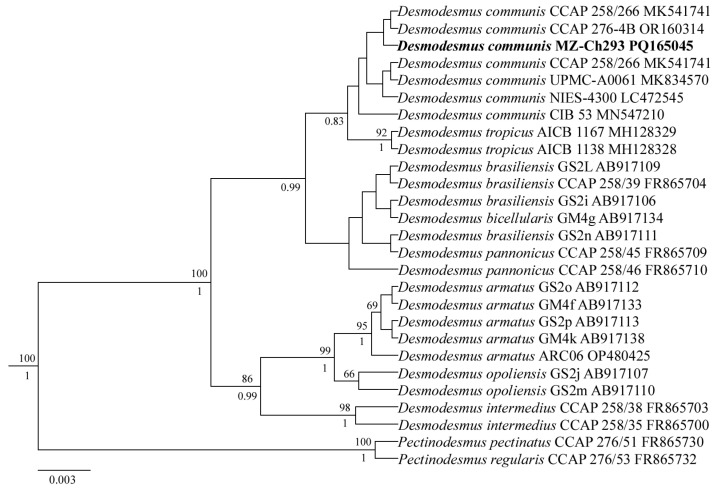
Bayesian inference (BI) tree for the strain *D. communis* MZ–Ch293 (indicated in bold) and other members of the green algal class Chlorophyceae inferred from 18S rRNA gene sequences (417 bp long). Numbers above branches indicate ML bootstrap proportions (>50%). Numbers under branches indicate Bayesian Markov chain Monte Carlo posterior probabilities > 0.8. The two *Pectinodesmus* species served as outgroup taxa.

Editing and assembling of the consensus sequences were carried out by comparing the direct and reverse chromatograms in Ridom TraceEdit ver. 1.1.0 and MEGA 7 [47]. The newly obtained sequence was supplemented with GenBank-extracted sequences for 24 *Desmodesmus* species and 2 outgroup taxa (taxa names and accession numbers are given in Figure 6). The alignment was automatically edited using the E-INS-i algorithm in MAFFT, ver. 7 [48] and then manually edited using MEGA 7 [47]. The resulting data set comprised 417 nucleotide sites for the nuclear 18S rRNA gene.

The 18S rDNA sequence data set was subjected to Bayesian inference (BI) and maximum likelihood (ML) analyses. The BI was performed using the software Beast ver. 1.10.1 software [49]. Model selection for the Bayesian approach was determined with jModelTest ver. 2.1.10 (Vigo, Spain) [50]. For Bayesian analysis, the K80 model was selected by the Bayesian information criterion. The Bayesian analysis was performed using four Markov chains and 7,000,000 generations sampling every 1000 generations, with the first 25% of sampled trees discarded. Posterior probabilities were then calculated from two independent runs using the 50% majority rule consensus of the kept trees. ML analyses were performed using RAxML, with 1000 bootstrap replicates [51]. Viewing and editing of trees were carried out with the software FigTree ver. 1.4.4 and Adobe Photoshop CC ver. 19.0.

### 4.2. Nanoparticles and Suspensions of Nanoparticles

CuO nanoparticles (Sigma-Aldrich, St. Louis, MO, USA) were used in this work. Before the experiment, the morphology and particle size were determined by scanning electron microscopy (SEM) on a high-resolution Merlin microscope (Carl Zeiss, Oberkochen, Germany) with an energy dispersion analyzer “10 mm^2^ SDD Detector—X-Act” (Oxford Instruments, Oxford, UK).

To introduce NPs into the cultivation medium, their aqueous dispersions were prepared. Particle powders (0.5 mg) were weighed using ViBRA HT analytical scales (Shinko Denshi, Tokyo, Japan), poured into Falcon tubes with screw caps of 10 mL with sterile distilled water (pH 7.1 ± 0.2) and mixed. After mixing, the suspensions were treated in an ultrasonic bath VBS-41H (ultrasonic power—180 W, volume—4 l, Vilitek, Moscow, Russia) for 15 min, involving 3 repetitions of 5 min with a minute interval to cool the dispersion. Thus, an aqueous colloidal solution with a concentration of 500 mg of NPs in 10 mL was obtained. After that, suspensions with a concentration of 50 micrograms in 10 mL and 5 mg in 10 mL were obtained from the initial solution by dilution.

To assess the stability of the obtained dispersed system (500 mg in 10 mL), the zeta potential of particles in suspensions was analyzed using a ZetasizerNanoZS analyzer (Malvern Instruments, Malvern, UK). The size of particles and aggregates of particles in the obtained colloidal solutions was determined by the method of dynamic light scattering on the ZetasizerNanoZS device.

### 4.3. Heavy Metals

Lead acetate (Pb(CH_3_COO)_2_) powders and cadmium sulfate (CdSO_4_) (Sigma-Aldrich, St. Louis, MO, USA) were used in this study. Their aqueous solutions based on sterile distilled water were prepared for the study. The initial concentrations of metals (Cd and Pb) were 5 and 50 mg L^−1^ for Cd and 50 and 500 mg L^−1^ for Pb. The working concentrations of metals were 0.1 and 1 mg L^−1^ for Cd and 1 and 10 mg L^−1^ for Pb.

### 4.4. The Effect of Pollutants on Microalgae

#### 4.4.1. Experiment Design

The BG-11 nutrient medium was used in the study. The initial optical density of the seeded algae test culture at a wavelength of 560 nm was 0.59. The introduction of nanoparticles and heavy metals was carried out immediately before the start of the experiment.

Six mL of nutrient medium containing microorganisms was introduced into each of the wells of the 6-well culture plate; 120 µL of suspensions of pollutants (NPs, heavy metals, and NPs + heavy metals) was also added and gently mixed in a circular motion; and 120 µL of distilled water was added to the control wells without the addition of NPs and heavy metals. Thus, during cultivation, microorganisms were exposed to the pollutant CuO-NPs (10, 100, and 1000 µg L^−1^), Cd (0.1 and 1 mg L^−1^), and Pb (1 and 10 mg L^−1^) in three replicates for each treatment and control in the nutrient medium BG-11. The concentrations of pollutants were chosen because these values lie within the limits of ecologically significant concentrations of pollutants [52,53]. Samples were taken on the first day (5 h of exposure) and the last day (14th day) of the experiment.

#### 4.4.2. Cell Viability Analysis

The main indicator of the state of the culture was the change in the % viability of the experimental test object. The control was the growth of algae in a clean environment without the addition of NPs and heavy metals. This indicator was determined using a Muse Cell Analyzer (Merck Millipore, Darmstadt, Germany) with a Muse Count & Viability reagent. Additionally, the total number of cells was determined in the presence of pollutants and control samples without the addition of NPs and heavy metals.

#### 4.4.3. Oxidative Stress Analysis

The degree of oxidative stress was also carried out using the Muse Cell Analyzer (Merck Millipore, Darmstadt, Germany) and a Muse Oxidative Stress kit.

#### 4.4.4. Determination of Pigment Content

To quantify the pigment composition of microalgae, the samples were extracted using dimethyl sulfoxide (DMSO) (Sigma-Aldrich, USA). To achieve this, 1 mL of the cell suspension was centrifuged for 5 min at 6000 revolutions on a MiniSpin centrifuge (Eppendorf, Hamburg, Germany). The supernatant was removed. The cells were incubated in DMSO at 70 °C for 10 min with intensive stirring. The cells were then precipitated by centrifugation.

The concentration of chlorophyll-*a* (Ch-*a*) and chlorophyll-b (Ch-*b*), as well as the total concentration of carotenoids (CRs) in the extract, was determined spectrophotometrically in 96-well plates using a Multiskan Sky microplate spectrophotometer (Thermo Scientific, Waltham, MA, USA). The calculation of the total concentration of carotenoids and the concentration of chlorophylls was carried out according to the equations [54]:

Chl-*a* = 13.34 D_666_ − 4.85 D_650_; (1)

Chl-*b* = 24.58 D_650_ − 6.65 D_666_; (2)

CR = (1000 D_480_ − 1.29 Chl-*a* − 53.76 Chl-*b*)/220 (3)

where Chl-*a*, Chl-*b*, and CR are concentrations of chlorophyll-a and -b and carotenoids in the extract, respectively (mg L^−1^), and D_λ_ is the optical density at the corresponding wavelength λ (nm).

#### 4.4.5. Bioaccumulation Analysis

The quantitative content of heavy metals in microalgae cells was determined by inductively coupled plasma atomic emission spectrometry (ICP-AES) on a Varian 720-ES ICP-AES spectrometer (Agilent Technologies, Santa Clara, CA, USA). During the preparation of samples for analysis, 2 mL was filtered through membrane filters (Millipore, Bedford, MA, USA) with a pore size of 0.45 microns. Microalgae incubated with nanoparticles and heavy metals at maximum concentrations were taken as samples for analysis. Samples for ICP-AES were prepared in accordance with the standard protocol [53].

#### 4.4.6. Statistical Analysis

All trials were conducted using 3–4 independent biological replicates with 3 to 6 technical replicates (depending on the analysis), which were then analyzed for significant differences (*p* < 0.05) against the control (zero-pollutant concentration) using a one-way Analysis of Variance test (ANOVA). The figures and tables show the average values and their standard deviations, indicating a statistically significant difference where applicable.

## Figures and Tables

**Table 1:** The content of heavy metals in *D. communis* cells at the end of the experiment (14 d) at maximum concentrations of pollutants in BG-11.

Treatments	Number of Cells *n* × 10^7^ mL^−1^	Heavy Metals Content, µg L^−1^	Heavy Metals Content by Cell, *n* × 10^−5^ µg L^−1^
Cd, 1 mg L^−1^	3.76	233	0.62
CuO-NPs + Cd, 1000 µg L^−1^ + 1 mg L^−1^	5.48	357	0.65
Pb, 10 mg L^−1^	2.5	441	1.76
CuO-NPs + Pb, 1000 µg L^−1^ + 1 mg L^−1^	4.11	1447	3.52

## Data Availability

Data are contained within the article.
